# Bis(μ-azido-κ^2^ *N* ^1^:*N* ^1^)bis­{(acetato-κ^2^ *O*,*O*′)[2,4,6-tris­(2-pyrid­yl)-1,3,5-triazine-κ^3^ *N* ^2^,*N* ^1^,*N* ^6^]lead(II)}

**DOI:** 10.1107/S1600536811038116

**Published:** 2011-09-30

**Authors:** Milad Dayani, Akbar Ghaemi, Seik Weng Ng, Edward R. T. Tiekink

**Affiliations:** a Young Researchers Club, Saveh Branch, Islamic Azad University, Saveh, Iran; b Department of Chemistry, Saveh Branch, Islamic Azad University, Saveh, Iran; c Department of Chemistry, University of Malaya, 50603 Kuala Lumpur, Malaysia; d Chemistry Department, Faculty of, Science, King Abdulaziz University, PO Box 80203 Jeddah, Saudi Arabia

## Abstract

The complete dinuclear title complex, [Pb_2_(C_2_H_3_O_2_)_2_(N_3_)_2_(C_18_H_12_N_6_)_2_], is generated by the application of a crystallographic centre of inversion. The Pb^II^ atom is coordinated by three N atoms of the tridentate ligand, two O atoms derived from an asymmetrically coordinating acetate ligand, and two azido-N atoms derived from two asymmetrically bridging azido ligands. The metal coordination geometry can be described as a square anti-prism with one position occupied by an unseen lone pair of electrons. In the ligand, the two coordinating pyridine rings are almost co-planar with the central pyrazine ring [dihedral angles = 0.47 (17) and 0.83 (18)°], but the terminal ring is twisted [dihedral angle = 19.76 (18)°]. In the crystal, the presence of π–π inter­actions [ring centroid distance between pyridyl rings = 3.581 (2) Å] leads to supra­molecular chains along the *a*-axis direction.

## Related literature

For related lead(II) complexes with the 2,4,6-tris­(2-pyrid­yl)-1,3,5-triazine ligand, see: Harrowfield *et al.* (1996**a* ▶,b* ▶, 2002 ▶).
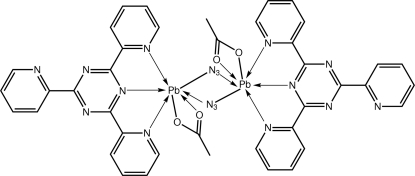


## Experimental

### 

#### Crystal data

[Pb_2_(C_2_H_3_O_2_)_2_(N_3_)_2_(C_18_H_12_N_6_)_2_]
*M* *_r_* = 1241.22
Triclinic, 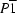

*a* = 8.5529 (4) Å
*b* = 11.0080 (5) Å
*c* = 11.8617 (5) Å
α = 86.739 (4)°
β = 70.928 (4)°
γ = 70.100 (4)°
*V* = 990.60 (8) Å^3^
*Z* = 1
Mo *K*α radiation
μ = 8.56 mm^−1^
*T* = 100 K
0.20 × 0.15 × 0.10 mm

#### Data collection

Agilent SuperNova Dual diffractometer with Atlas detector
Absorption correction: multi-scan (*CrysAlis PRO*; Agilent, 2010 ▶) *T*_min_ = 0.279, *T*_max_ = 0.482
7814 measured reflections
4358 independent reflections
4046 reflections with *I* > 2σ(*I*)
*R*_int_ = 0.059

#### Refinement

*R*[*F* ^2^ > 2σ(*F* ^2^)] = 0.024
*wR*(*F* ^2^) = 0.049
*S* = 0.97
4358 reflections
290 parameters
H-atom parameters constrained
Δρ_max_ = 2.01 e Å^−3^
Δρ_min_ = −2.14 e Å^−3^

### 

Data collection: *CrysAlis PRO* (Agilent, 2010 ▶); cell refinement: *CrysAlis PRO*; data reduction: *CrysAlis PRO*; program(s) used to solve structure: *SHELXS97* (Sheldrick, 2008 ▶); program(s) used to refine structure: *SHELXL97* (Sheldrick, 2008 ▶); molecular graphics: *ORTEP-3* (Farrugia, 1997 ▶) and *DIAMOND* (Brandenburg, 2006 ▶); software used to prepare material for publication: *publCIF* (Westrip, 2010 ▶).

## Supplementary Material

Crystal structure: contains datablock(s) general, I. DOI: 10.1107/S1600536811038116/hb6410sup1.cif

Structure factors: contains datablock(s) I. DOI: 10.1107/S1600536811038116/hb6410Isup2.hkl

Additional supplementary materials:  crystallographic information; 3D view; checkCIF report

## Figures and Tables

**Table 1:** Selected bond lengths (Å)

Pb—O1	2.349 (3)
Pb—O2	2.550 (3)
Pb—N1	2.684 (3)
Pb—N2	2.698 (3)
Pb—N6	2.702 (3)
Pb—N7	2.586 (3)
Pb—N7^i^	2.874 (3)

Symmetry code: (i) 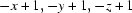.

**Fig. 1.**
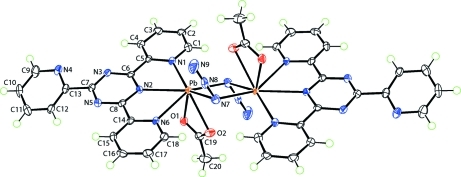
The molecular structure of (I) showing displacement ellipsoids at the 50% probability level. Unlabelled atoms are generated by the symmetry operation 1 - x, 1 - y, 1 - z.

**Fig. 2.**
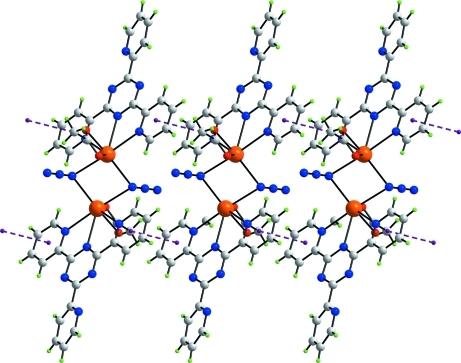
Supramolecular chains in (I) mediated by π···π interactions shown as purple dashed lines.

**Fig. 3.**
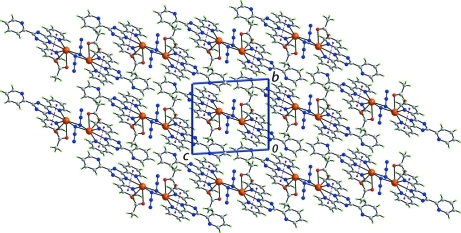
A view of the unit-cell contents of (I) in projection down the a axis. The π···π interactions are shown as purple dashed lines.

## supplementary crystallographic information

### Comment

Lead(II) complexes of the 2,4,6-tris(2-pyridyl)-1,3,5-triazine ligand (tptz) have been reported in the literature (Harrowfield *et al.* 1996*a*; Harrowfield *et al.* 1996*b*; Harrowfield *et al.* 2002). In each of the nine known structures, the tptz ligand has been shown to function as a tridentate ligand as observed in the dinuclear structure of the title compound, (I).

The complete dinuclear molecule of (I) is generated by the application of a centre of inversion. The Pb^II^ atom is seven coordinate within a N_5_O_2_ donor set defined by three N atoms of the tptz ligand, two atoms derived from two µ-azido anions, and two O atoms derived from an asymmetrically chelating acetate, Table 1. The coordination geometry is based on a square anti-prism with one face defined by the O1, O2, N2 and N6 atoms, and the other by the N1, N7, N7^i^ and the lone pair of electrons; symmetry operation *i*: 1 - *x*, 1 - *y*, -*z*. The µ-azido bridge is non-symmetric, Table 1, leading the Pb_2_N_2_ central ring to have the shape of a trapezium. Within the tptz ligand, the two coordinating pyridine rings are co-planar with the central pyrazine ring (r.m.s. deviation = 0.016 Å), forming dihedral angles of 0.47 (17) (N1-pyridine) and 0.83 (18)° (N6-pyridine) but, the N4-pyridine ring is twisted out of the plane of the central triazine [dihedral angle = 19.76 (18)°].

The most prominent feature of the crystal packing is the formation of π···π interactions. These occur between the translationally related N1- and N4-pyridine rings with the separation between the ring centroids being 3.581 (2) Å for symmetry operation *x* - 1, *y*, *z*. These lead to the formation of supramolecular chains along the *a* axis. Chains assemble into layers that stack along the *b*-direction Fig. 3.

### Experimental

The title complex was synthesized by the addition of 2,4,6-tris(2-pyridyl)-1,3,5-triazine (tptz; 0.312 g, 1 mmol) to a solution of lead(II) acetate trihydrate (0.378 g, 1 mmol) in 10 ml of DMF, followed by the drop wise addition of sodium azide (0.065 g, 1 mmol) dissolved in a minimum volume of water. After stirring for 2 h, the reaction solution was filtered. The resulting clear yellow solution left to stand in air. After 4 days, yellow prisms were obtained; Yield: 70%; *M*.pt. 540–542 K.

### Refinement

The H-atoms were placed in calculated positions (C—H 0.95 to 0.981164 Å) and were included in the refinement in the riding model approximation, with *U*_iso_(H) set to 1.2 to 1.5*U*_equiv_(C). The maximum and minimum residual electron density peaks of 2.01 and 2.14 e Å^-3^, respectively, were located 0.93 Å and 0.55 Å from the Pb atom.

### Crystal data

**Table:**

[Pb_2_(C_2_H_3_O_2_)_2_(N_3_)_2_(C_18_H_12_N_6_)_2_]	*Z* = 1
*M**_r_* = 1241.22	*F*(000) = 592
Triclinic, *P*1	*D*_x_ = 2.081 Mg m^−^^3^
Hall symbol: -P 1	Mo *K*α radiation, λ = 0.71073 Å
*a* = 8.5529 (4) Å	Cell parameters from 5891 reflections
*b* = 11.0080 (5) Å	θ = 2.6–29.2°
*c* = 11.8617 (5) Å	µ = 8.56 mm^−^^1^
α = 86.739 (4)°	*T* = 100 K
β = 70.928 (4)°	Prism, yellow
γ = 70.100 (4)°	0.20 × 0.15 × 0.10 mm
*V* = 990.60 (8) Å^3^	

### Data collection

**Table:**

Agilent SuperNova Dual diffractometer with Atlas detector	4358 independent reflections
Radiation source: SuperNova (Mo) X-ray Source	4046 reflections with *I* > 2σ(*I*)
Mirror	*R*_int_ = 0.059
Detector resolution: 10.4041 pixels mm^-1^	θ_max_ = 27.5°, θ_min_ = 2.6°
ω scan	*h* = −10→9
Absorption correction: multi-scan (*CrysAlis PRO*; Agilent, 2010)	*k* = −13→10
*T*_min_ = 0.279, *T*_max_ = 0.482	*l* = −13→14
7814 measured reflections	

### Refinement

**Table:**

Refinement on *F*^2^	Primary atom site location: structure-invariant direct methods
Least-squares matrix: full	Secondary atom site location: difference Fourier map
*R*[*F*^2^ > 2σ(*F*^2^)] = 0.024	Hydrogen site location: inferred from neighbouring sites
*wR*(*F*^2^) = 0.049	H-atom parameters constrained
*S* = 0.97	*w* = 1/[σ^2^(*F*_o_^2^) + (0.0106*P*)^2^] where *P* = (*F*_o_^2^ + 2*F*_c_^2^)/3
4358 reflections	(Δ/σ)_max_ = 0.002
290 parameters	Δρ_max_ = 2.01 e Å^−^^3^
0 restraints	Δρ_min_ = −2.14 e Å^−^^3^

### Special details

**Table:**

Geometry. All e.s.d.'s (except the e.s.d. in the dihedral angle between two l.s. planes) are estimated using the full covariance matrix. The cell e.s.d.'s are taken into account individually in the estimation of e.s.d.'s in distances, angles and torsion angles; correlations between e.s.d.'s in cell parameters are only used when they are defined by crystal symmetry. An approximate (isotropic) treatment of cell e.s.d.'s is used for estimating e.s.d.'s involving l.s. planes.
Refinement. Refinement of *F*^2^ against ALL reflections. The weighted *R*-factor *wR* and goodness of fit *S* are based on *F*^2^, conventional *R*-factors *R* are based on *F*, with *F* set to zero for negative *F*^2^. The threshold expression of *F*^2^ > σ(*F*^2^) is used only for calculating *R*-factors(gt) *etc*. and is not relevant to the choice of reflections for refinement. *R*-factors based on *F*^2^ are statistically about twice as large as those based on *F*, and *R*- factors based on ALL data will be even larger.

### Fractional atomic coordinates and isotropic or equivalent isotropic displacement parameters (Å^2^)

**Table:**

	*x*	*y*	*z*	*U*_iso_*/*U*_eq_	
Pb	0.612101 (15)	0.416726 (12)	0.347893 (11)	0.01042 (5)	
O1	0.5120 (3)	0.5621 (2)	0.2132 (2)	0.0151 (6)	
O2	0.5432 (3)	0.6610 (2)	0.3570 (2)	0.0167 (6)	
N1	0.4835 (3)	0.2650 (3)	0.2624 (3)	0.0128 (7)	
N2	0.7962 (3)	0.2875 (3)	0.1327 (3)	0.0103 (6)	
N3	0.8279 (4)	0.1391 (3)	−0.0182 (3)	0.0119 (6)	
N4	1.0363 (4)	−0.0059 (3)	−0.2293 (3)	0.0149 (7)	
N5	1.0514 (4)	0.2285 (3)	−0.0385 (3)	0.0129 (7)	
N6	0.9239 (3)	0.4423 (3)	0.2205 (3)	0.0111 (6)	
N7	0.2803 (4)	0.5086 (3)	0.4691 (3)	0.0162 (7)	
N8	0.1867 (4)	0.6149 (3)	0.4603 (3)	0.0153 (7)	
N9	0.0947 (4)	0.7192 (4)	0.4520 (3)	0.0276 (9)	
C1	0.3281 (5)	0.2554 (4)	0.3282 (3)	0.0159 (8)	
H1	0.2676	0.3031	0.4032	0.019*	
C2	0.2523 (4)	0.1794 (4)	0.2918 (3)	0.0156 (8)	
H2	0.1421	0.1750	0.3414	0.019*	
C3	0.3376 (4)	0.1098 (4)	0.1829 (3)	0.0157 (8)	
H3	0.2860	0.0586	0.1554	0.019*	
C4	0.5005 (4)	0.1162 (4)	0.1146 (3)	0.0132 (8)	
H4	0.5642	0.0678	0.0401	0.016*	
C5	0.5687 (4)	0.1949 (3)	0.1572 (3)	0.0103 (7)	
C6	0.7406 (4)	0.2074 (3)	0.0872 (3)	0.0104 (7)	
C7	0.9812 (4)	0.1554 (3)	−0.0777 (3)	0.0105 (7)	
C8	0.9539 (4)	0.2935 (3)	0.0672 (3)	0.0106 (7)	
C9	1.1213 (4)	−0.0594 (4)	−0.3404 (4)	0.0200 (9)	
H9	1.0950	−0.1299	−0.3625	0.024*	
C10	1.2461 (5)	−0.0187 (4)	−0.4259 (3)	0.0211 (9)	
H10	1.3009	−0.0591	−0.5043	0.025*	
C11	1.2887 (5)	0.0824 (4)	−0.3942 (3)	0.0199 (9)	
H11	1.3734	0.1128	−0.4502	0.024*	
C12	1.2045 (4)	0.1380 (4)	−0.2787 (3)	0.0148 (8)	
H12	1.2320	0.2062	−0.2533	0.018*	
C13	1.0793 (4)	0.0921 (4)	−0.2008 (3)	0.0135 (8)	
C14	1.0237 (4)	0.3786 (3)	0.1128 (3)	0.0108 (7)	
C15	1.1825 (4)	0.3928 (4)	0.0472 (3)	0.0127 (8)	
H15	1.2490	0.3480	−0.0287	0.015*	
C16	1.2428 (5)	0.4728 (4)	0.0937 (3)	0.0154 (8)	
H16	1.3515	0.4840	0.0502	0.018*	
C17	1.1433 (4)	0.5364 (4)	0.2043 (3)	0.0152 (8)	
H17	1.1832	0.5910	0.2387	0.018*	
C18	0.9848 (4)	0.5194 (4)	0.2640 (3)	0.0138 (8)	
H18	0.9158	0.5646	0.3394	0.017*	
C19	0.4957 (4)	0.6651 (4)	0.2671 (3)	0.0145 (8)	
C20	0.4162 (5)	0.7942 (4)	0.2216 (4)	0.0216 (9)	
H20A	0.3924	0.7807	0.1488	0.032*	
H20B	0.4985	0.8421	0.2038	0.032*	
H20C	0.3059	0.8439	0.2827	0.032*	

### Atomic displacement parameters (Å^2^)

**Table:**

	*U*^11^	*U*^22^	*U*^33^	*U*^12^	*U*^13^	*U*^23^
Pb	0.00968 (8)	0.01033 (8)	0.01004 (8)	−0.00308 (5)	−0.00182 (5)	−0.00064 (6)
O1	0.0109 (12)	0.0159 (15)	0.0157 (14)	−0.0040 (10)	−0.0009 (10)	−0.0023 (11)
O2	0.0180 (13)	0.0141 (15)	0.0211 (15)	−0.0073 (11)	−0.0089 (11)	0.0025 (12)
N1	0.0102 (14)	0.0105 (17)	0.0141 (16)	−0.0015 (12)	−0.0011 (12)	−0.0009 (13)
N2	0.0092 (14)	0.0108 (16)	0.0097 (15)	−0.0031 (12)	−0.0019 (12)	0.0003 (13)
N3	0.0117 (15)	0.0092 (16)	0.0134 (16)	−0.0021 (12)	−0.0036 (12)	−0.0010 (13)
N4	0.0108 (15)	0.0140 (18)	0.0178 (17)	−0.0007 (12)	−0.0048 (13)	−0.0039 (14)
N5	0.0117 (15)	0.0122 (17)	0.0123 (16)	−0.0030 (12)	−0.0019 (12)	−0.0003 (13)
N6	0.0089 (14)	0.0087 (16)	0.0123 (16)	−0.0008 (12)	−0.0013 (12)	−0.0013 (13)
N7	0.0136 (15)	0.0168 (19)	0.0169 (17)	−0.0053 (13)	−0.0032 (13)	0.0007 (14)
N8	0.0118 (15)	0.023 (2)	0.0099 (16)	−0.0070 (14)	0.0002 (12)	−0.0050 (14)
N9	0.0220 (18)	0.024 (2)	0.026 (2)	0.0037 (15)	−0.0051 (15)	−0.0040 (17)
C1	0.0147 (18)	0.016 (2)	0.0143 (19)	−0.0052 (15)	−0.0012 (15)	−0.0007 (16)
C2	0.0100 (17)	0.012 (2)	0.022 (2)	−0.0060 (14)	0.0004 (15)	0.0020 (16)
C3	0.0136 (18)	0.014 (2)	0.021 (2)	−0.0081 (15)	−0.0048 (15)	0.0011 (17)
C4	0.0141 (18)	0.012 (2)	0.0122 (19)	−0.0044 (14)	−0.0033 (15)	−0.0001 (15)
C5	0.0090 (16)	0.0063 (18)	0.0130 (18)	−0.0001 (13)	−0.0027 (14)	−0.0002 (15)
C6	0.0075 (16)	0.0062 (18)	0.0148 (19)	−0.0004 (13)	−0.0024 (14)	0.0024 (15)
C7	0.0057 (16)	0.0087 (19)	0.0138 (19)	0.0017 (13)	−0.0033 (14)	0.0009 (15)
C8	0.0100 (17)	0.0076 (19)	0.0124 (18)	−0.0001 (13)	−0.0044 (14)	0.0013 (15)
C9	0.0124 (18)	0.023 (2)	0.023 (2)	−0.0042 (16)	−0.0041 (16)	−0.0084 (18)
C10	0.0152 (19)	0.029 (3)	0.012 (2)	−0.0018 (17)	0.0005 (15)	−0.0078 (18)
C11	0.0139 (18)	0.026 (2)	0.014 (2)	−0.0057 (16)	0.0010 (15)	0.0007 (18)
C12	0.0119 (17)	0.012 (2)	0.016 (2)	−0.0013 (14)	−0.0021 (15)	−0.0034 (16)
C13	0.0089 (17)	0.012 (2)	0.0154 (19)	0.0011 (14)	−0.0024 (14)	−0.0025 (16)
C14	0.0104 (16)	0.0072 (18)	0.0120 (18)	0.0010 (13)	−0.0045 (14)	0.0010 (15)
C15	0.0098 (17)	0.014 (2)	0.0107 (18)	−0.0008 (14)	−0.0015 (14)	−0.0012 (15)
C16	0.0128 (18)	0.015 (2)	0.019 (2)	−0.0075 (15)	−0.0051 (15)	0.0063 (17)
C17	0.0136 (18)	0.016 (2)	0.020 (2)	−0.0092 (15)	−0.0069 (15)	0.0014 (16)
C18	0.0157 (18)	0.012 (2)	0.0123 (19)	−0.0037 (15)	−0.0041 (15)	0.0003 (15)
C19	0.0066 (16)	0.013 (2)	0.021 (2)	−0.0048 (14)	−0.0007 (15)	0.0016 (16)
C20	0.019 (2)	0.018 (2)	0.028 (2)	−0.0049 (16)	−0.0121 (17)	0.0085 (18)

### Geometric parameters (Å, °)

**Table:**

Pb—O1	2.349 (3)	C3—C4	1.388 (5)
Pb—O2	2.550 (3)	C3—H3	0.9500
Pb—N1	2.684 (3)	C4—C5	1.390 (5)
Pb—N2	2.698 (3)	C4—H4	0.9500
Pb—N6	2.702 (3)	C5—C6	1.483 (5)
Pb—N7	2.586 (3)	C7—C13	1.495 (5)
Pb—N7^i^	2.874 (3)	C8—C14	1.475 (5)
O1—C19	1.277 (4)	C9—C10	1.391 (5)
O2—C19	1.252 (4)	C9—H9	0.9500
N1—C1	1.339 (4)	C10—C11	1.388 (6)
N1—C5	1.347 (4)	C10—H10	0.9500
N2—C8	1.341 (4)	C11—C12	1.386 (5)
N2—C6	1.342 (5)	C11—H11	0.9500
N3—C7	1.338 (4)	C12—C13	1.387 (5)
N3—C6	1.341 (4)	C12—H12	0.9500
N4—C9	1.335 (5)	C14—C15	1.384 (5)
N4—C13	1.345 (5)	C15—C16	1.377 (5)
N5—C7	1.334 (5)	C15—H15	0.9500
N5—C8	1.339 (4)	C16—C17	1.380 (5)
N6—C18	1.335 (5)	C16—H16	0.9500
N6—C14	1.354 (4)	C17—C18	1.381 (5)
N7—N8	1.193 (4)	C17—H17	0.9500
N8—N9	1.170 (5)	C18—H18	0.9500
C1—C2	1.379 (5)	C19—C20	1.506 (5)
C1—H1	0.9500	C20—H20A	0.9800
C2—C3	1.380 (5)	C20—H20B	0.9800
C2—H2	0.9500	C20—H20C	0.9800
			
O1—Pb—O2	53.37 (9)	N3—C6—N2	124.8 (3)
O1—Pb—N7	80.25 (9)	N3—C6—C5	117.3 (3)
O2—Pb—N7	76.00 (9)	N2—C6—C5	117.8 (3)
O1—Pb—N1	83.67 (9)	N5—C7—N3	125.3 (3)
O2—Pb—N1	132.61 (8)	N5—C7—C13	116.6 (3)
N7—Pb—N1	78.04 (9)	N3—C7—C13	118.1 (3)
O1—Pb—N2	76.16 (9)	N2—C8—N5	124.0 (3)
O2—Pb—N2	117.28 (9)	N2—C8—C14	118.3 (3)
N7—Pb—N2	133.65 (9)	N5—C8—C14	117.7 (3)
N1—Pb—N2	60.27 (8)	N4—C9—C10	124.2 (4)
O1—Pb—N6	82.58 (8)	N4—C9—H9	117.9
O2—Pb—N6	76.90 (8)	C10—C9—H9	117.9
N7—Pb—N6	152.86 (10)	C11—C10—C9	118.5 (3)
N1—Pb—N6	120.73 (8)	C11—C10—H10	120.7
N2—Pb—N6	60.46 (9)	C9—C10—H10	120.7
O1—Pb—C19	26.90 (10)	C12—C11—C10	118.3 (4)
O2—Pb—C19	26.52 (9)	C12—C11—H11	120.9
N7—Pb—C19	75.47 (10)	C10—C11—H11	120.9
N1—Pb—C19	108.20 (10)	C11—C12—C13	118.8 (4)
N2—Pb—C19	97.94 (10)	C11—C12—H12	120.6
N6—Pb—C19	79.70 (9)	C13—C12—H12	120.6
C19—O1—Pb	96.8 (2)	N4—C13—C12	124.0 (3)
C19—O2—Pb	88.1 (2)	N4—C13—C7	116.6 (3)
C1—N1—C5	117.7 (3)	C12—C13—C7	119.4 (3)
C1—N1—Pb	118.8 (2)	N6—C14—C15	122.3 (3)
C5—N1—Pb	123.5 (2)	N6—C14—C8	116.7 (3)
C8—N2—C6	115.6 (3)	C15—C14—C8	121.0 (3)
C8—N2—Pb	122.1 (2)	C16—C15—C14	119.0 (3)
C6—N2—Pb	122.2 (2)	C16—C15—H15	120.5
C7—N3—C6	114.6 (3)	C14—C15—H15	120.5
C9—N4—C13	116.2 (3)	C17—C16—C15	119.1 (3)
C7—N5—C8	115.6 (3)	C17—C16—H16	120.4
C18—N6—C14	117.7 (3)	C15—C16—H16	120.4
C18—N6—Pb	119.9 (2)	C16—C17—C18	118.8 (3)
C14—N6—Pb	122.4 (2)	C16—C17—H17	120.6
N8—N7—Pb	123.7 (2)	C18—C17—H17	120.6
N9—N8—N7	179.9 (5)	N6—C18—C17	123.0 (3)
N1—C1—C2	122.9 (3)	N6—C18—H18	118.5
N1—C1—H1	118.6	C17—C18—H18	118.5
C2—C1—H1	118.6	O2—C19—O1	121.5 (3)
C1—C2—C3	119.6 (3)	O2—C19—C20	119.5 (3)
C1—C2—H2	120.2	O1—C19—C20	119.0 (3)
C3—C2—H2	120.2	O2—C19—Pb	65.4 (2)
C2—C3—C4	118.4 (3)	O1—C19—Pb	56.31 (19)
C2—C3—H3	120.8	C20—C19—Pb	173.3 (3)
C4—C3—H3	120.8	C19—C20—H20A	109.5
C3—C4—C5	118.7 (3)	C19—C20—H20B	109.5
C3—C4—H4	120.6	H20A—C20—H20B	109.5
C5—C4—H4	120.6	C19—C20—H20C	109.5
N1—C5—C4	122.8 (3)	H20A—C20—H20C	109.5
N1—C5—C6	116.1 (3)	H20B—C20—H20C	109.5
C4—C5—C6	121.1 (3)		
			
O2—Pb—O1—C19	−2.67 (18)	C3—C4—C5—C6	−178.9 (3)
N7—Pb—O1—C19	77.06 (19)	C7—N3—C6—N2	0.5 (5)
N1—Pb—O1—C19	155.96 (19)	C7—N3—C6—C5	179.3 (3)
N2—Pb—O1—C19	−143.2 (2)	C8—N2—C6—N3	−2.4 (5)
N6—Pb—O1—C19	−81.84 (19)	Pb—N2—C6—N3	−178.8 (3)
O1—Pb—O2—C19	2.71 (18)	C8—N2—C6—C5	178.8 (3)
N7—Pb—O2—C19	−85.5 (2)	Pb—N2—C6—C5	2.3 (4)
N1—Pb—O2—C19	−26.8 (2)	N1—C5—C6—N3	−179.7 (3)
N2—Pb—O2—C19	46.8 (2)	C4—C5—C6—N3	−0.5 (5)
N6—Pb—O2—C19	93.2 (2)	N1—C5—C6—N2	−0.7 (5)
O1—Pb—N1—C1	−102.3 (3)	C4—C5—C6—N2	178.5 (3)
O2—Pb—N1—C1	−78.9 (3)	C8—N5—C7—N3	−2.8 (5)
N7—Pb—N1—C1	−20.9 (3)	C8—N5—C7—C13	174.2 (3)
N2—Pb—N1—C1	−179.9 (3)	C6—N3—C7—N5	2.3 (5)
N6—Pb—N1—C1	−179.7 (2)	C6—N3—C7—C13	−174.7 (3)
C19—Pb—N1—C1	−91.1 (3)	C6—N2—C8—N5	1.8 (5)
O1—Pb—N1—C5	79.2 (3)	Pb—N2—C8—N5	178.3 (3)
O2—Pb—N1—C5	102.6 (3)	C6—N2—C8—C14	−179.3 (3)
N7—Pb—N1—C5	160.6 (3)	Pb—N2—C8—C14	−2.8 (4)
N2—Pb—N1—C5	1.6 (3)	C7—N5—C8—N2	0.6 (5)
N6—Pb—N1—C5	1.8 (3)	C7—N5—C8—C14	−178.3 (3)
C19—Pb—N1—C5	90.4 (3)	C13—N4—C9—C10	−1.1 (6)
O1—Pb—N2—C8	91.2 (3)	N4—C9—C10—C11	1.3 (6)
O2—Pb—N2—C8	56.1 (3)	C9—C10—C11—C12	−0.1 (6)
N7—Pb—N2—C8	152.8 (2)	C10—C11—C12—C13	−1.2 (6)
N1—Pb—N2—C8	−178.2 (3)	C9—N4—C13—C12	−0.3 (5)
N6—Pb—N2—C8	2.0 (2)	C9—N4—C13—C7	178.3 (3)
C19—Pb—N2—C8	75.3 (3)	C11—C12—C13—N4	1.5 (6)
O1—Pb—N2—C6	−92.5 (3)	C11—C12—C13—C7	−177.0 (3)
O2—Pb—N2—C6	−127.6 (3)	N5—C7—C13—N4	163.6 (3)
N7—Pb—N2—C6	−31.0 (3)	N3—C7—C13—N4	−19.2 (5)
N1—Pb—N2—C6	−2.0 (3)	N5—C7—C13—C12	−17.7 (5)
N6—Pb—N2—C6	178.2 (3)	N3—C7—C13—C12	159.5 (3)
C19—Pb—N2—C6	−108.4 (3)	C18—N6—C14—C15	−1.0 (5)
O1—Pb—N6—C18	101.2 (3)	Pb—N6—C14—C15	179.4 (3)
O2—Pb—N6—C18	47.2 (3)	C18—N6—C14—C8	179.7 (3)
N7—Pb—N6—C18	50.1 (4)	Pb—N6—C14—C8	0.1 (4)
N1—Pb—N6—C18	179.2 (2)	N2—C8—C14—N6	1.8 (5)
N2—Pb—N6—C18	179.4 (3)	N5—C8—C14—N6	−179.2 (3)
C19—Pb—N6—C18	74.1 (3)	N2—C8—C14—C15	−177.5 (3)
O1—Pb—N6—C14	−79.2 (3)	N5—C8—C14—C15	1.4 (5)
O2—Pb—N6—C14	−133.2 (3)	N6—C14—C15—C16	1.0 (5)
N7—Pb—N6—C14	−130.3 (3)	C8—C14—C15—C16	−179.7 (3)
N1—Pb—N6—C14	−1.2 (3)	C14—C15—C16—C17	0.0 (6)
N2—Pb—N6—C14	−1.0 (2)	C15—C16—C17—C18	−1.0 (6)
C19—Pb—N6—C14	−106.3 (3)	C14—N6—C18—C17	−0.1 (5)
O1—Pb—N7—N8	−28.8 (3)	Pb—N6—C18—C17	179.6 (3)
O2—Pb—N7—N8	25.6 (3)	C16—C17—C18—N6	1.1 (6)
N1—Pb—N7—N8	−114.4 (3)	Pb—O2—C19—O1	−4.7 (3)
N2—Pb—N7—N8	−88.9 (3)	Pb—O2—C19—C20	174.9 (3)
N6—Pb—N7—N8	22.7 (4)	Pb—O1—C19—O2	5.1 (3)
C19—Pb—N7—N8	−1.7 (3)	Pb—O1—C19—C20	−174.4 (3)
C5—N1—C1—C2	−1.1 (5)	O1—Pb—C19—O2	−175.2 (3)
Pb—N1—C1—C2	−179.7 (3)	N7—Pb—C19—O2	87.7 (2)
N1—C1—C2—C3	−0.3 (6)	N1—Pb—C19—O2	159.58 (18)
C1—C2—C3—C4	1.6 (6)	N2—Pb—C19—O2	−139.18 (19)
C2—C3—C4—C5	−1.6 (5)	N6—Pb—C19—O2	−81.28 (19)
C1—N1—C5—C4	1.1 (5)	O2—Pb—C19—O1	175.2 (3)
Pb—N1—C5—C4	179.6 (3)	N7—Pb—C19—O1	−97.13 (19)
C1—N1—C5—C6	−179.7 (3)	N1—Pb—C19—O1	−25.2 (2)
Pb—N1—C5—C6	−1.2 (4)	N2—Pb—C19—O1	36.01 (19)
C3—C4—C5—N1	0.2 (6)	N6—Pb—C19—O1	93.91 (19)

Symmetry codes: (i) −*x*+1, −*y*+1, −*z*+1.
